# A novel *TSC2* c.4511 T > C missense variant associated with tuberous sclerosis complex

**DOI:** 10.1186/s12881-020-01120-z

**Published:** 2020-09-11

**Authors:** Shunzhi He, Na Lv, Hongchu Bao, Xiong Wang, Jing Li

**Affiliations:** 1 Reproductive Medicine Center, The Affiliated Yantai Yuhuangding Hospital of Qingdao University, Yantai, Shandong 264000 P.R. China; 2 Department of Prenatal Diagnosis, The Affiliated Yantai Yuhuangding Hospital of Qingdao University, Yantai, Shandong 264000 P.R. China; 3 Electrocardiogram Room, The Affiliated Yantai Yuhuangding Hospital of Qingdao University, Yantai, Shandong 264000 P.R. China

**Keywords:** Tuberous sclerosis complex, *TSC1*, *TSC2*, Novel variant, Next generation sequencing, Preimplantation genetic testing

## Abstract

**Background:**

Tuberous sclerosis complex (TSC) is an autosomal-dominant hereditary disease characterized by hamartomas of multiple organ systems, including the brain, skin, heart, kidney and lung. Genetically, TSC is caused by pathogenic variants in the *TSC1* or *TSC2* gene.

**Case presentation:**

We reported a sporadic case of a 32-year-old Han Chinese male diagnosed with TSC, whose spouse had a history of two spontaneous miscarriages and an induced abortion of a 30-week fetus identified with cardiac rhabdomyoma by ultrasound. A novel heterozygous missense variant in the *TSC2* gene (Exon35:c.4511 T > C:p.L1504P) was identified in the male patient and the aborted fetus by next-generation sequencing, but not in his wife or both his parents. According to the ACMG/AMP criteria, this variant was classified as a “likely pathogenic” variant.

**Conclusion:**

The novel *TSC2*:c.4511 T > C variant identified was highly likely associated with TSC and could potentially lead to adverse reproductive outcomes. IVF-ET and pre-implantation genetic diagnosis for TSC are recommended for this patient in the future to prevent fetal TSC.

## Background

Tuberous sclerosis complex (TSC) is an autosomal dominant hereditary disease that is characterized by hamartomas of multiple organ systems, including the brain, skin, heart, kidney and lung. Symptoms of the central nervous system include epilepsy, learning disabilities, behavioural problems, and autism. Additionally, 70–80% of TSC patients develop kidney manifestations, usually angiomyolipoma and renal cysts. Genetically, TSC is caused by pathogenic variants in the *TSC1* or *TSC2* gene [1], accounting for approximately 10–30% and nearly 70% of all cases respectively [2]. Variants in *TSC2* cause more severe symptoms [3]. In this study, we identified a novel de novo variant in *TSC2* in a male TSC patient and his aborted 30-week fetus with cardiac rhabdomyoma by ultrasonography, a typical symptom of fetal TSC.

## Case presentation

The patient (proband) was a 32-year-old male, who was admitted to our reproductive centre with a referral for “recurrent spontaneous miscarriage of his spouse”. He had a history of head injury at the age of 6 years old, accompanied by brief loss of consciousness, but no skull fracture or intracranial haemorrhage occurred. One year after, fibrous nodules gradually appeared on the tip of his nose, nasolabial groove, cheek and mandible. He started to experience recurring epilepsy more than 10 attacks a year, which manifested as loss of consciousness, spastic convulsions of the limbs, eyes-rolling, occasional foaming at the mouth and biting of the tongue and lip, no loss of bowel or bladder control. The frequency of epilepsy attacks gradually decreased after the patient was 14 years old. At the age of 18, the patient underwent laser treatment for facial nodules so the facial nodules are currently atypical. The growth, development and intelligence of the paternal sample was basically unaffected by the disease. The patient denied any histories of other chronic diseases, smoking or drinking. His parents were healthy without any family history.

**Fig. 1:**
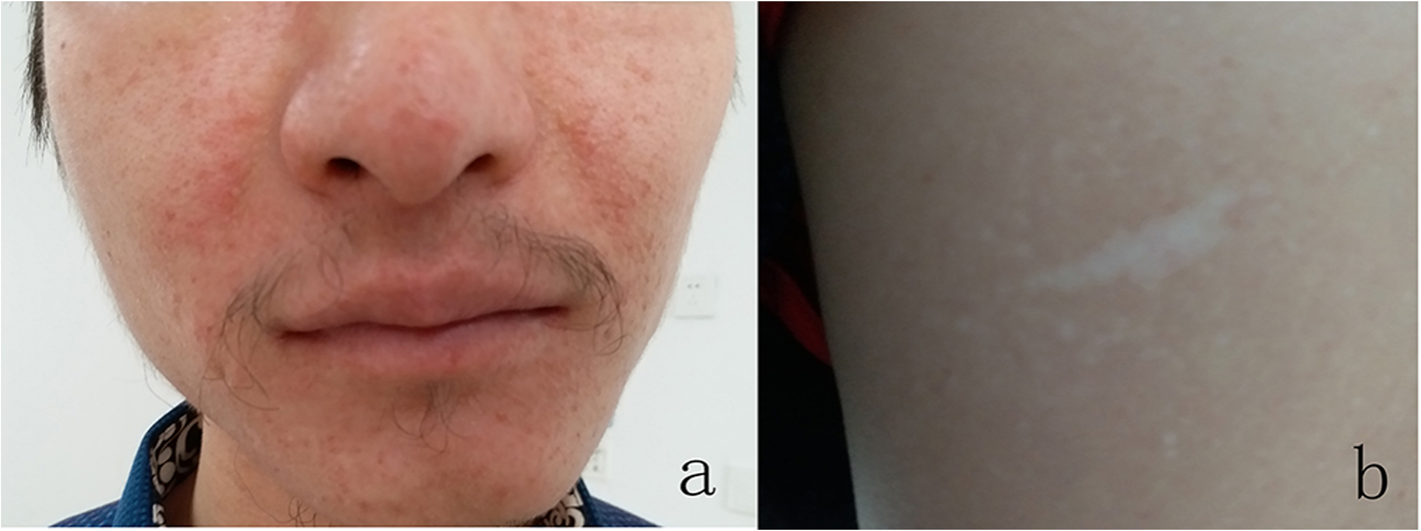
Photographs of the paternal sample showing (**a**) facial angiofibromas and (**b**) hypopigmented macules on the back

**Fig. 2:**
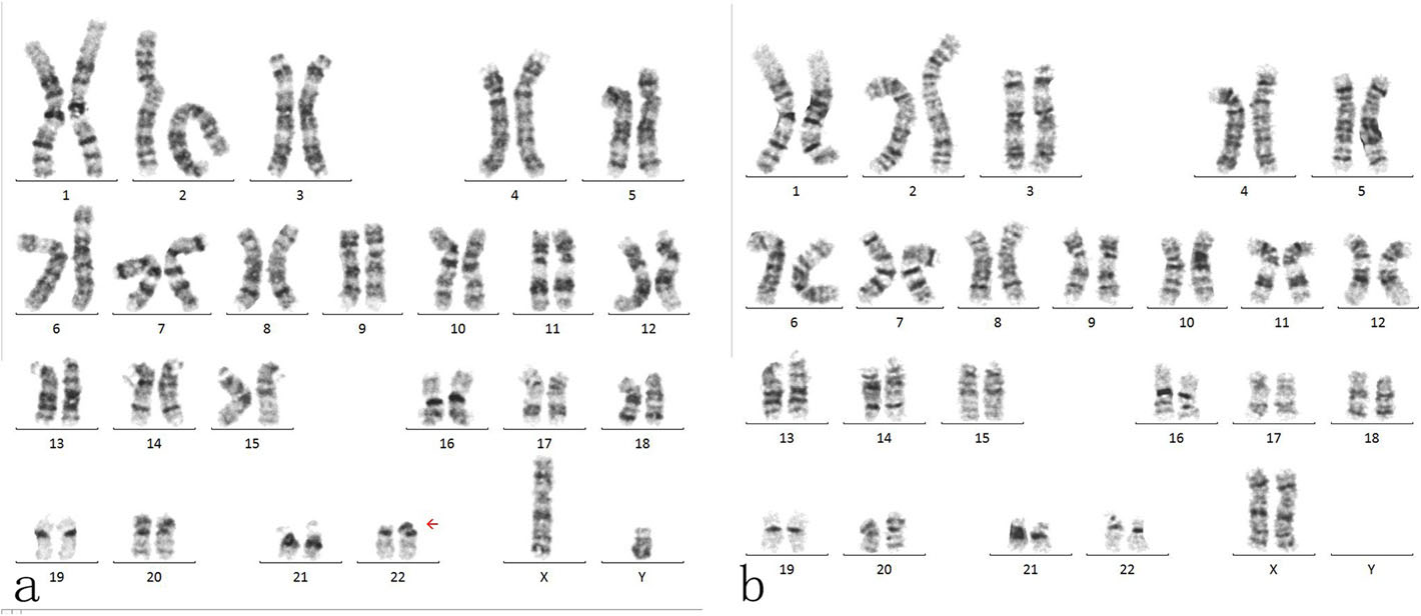
Chromosomal analysis results for (**a**) the paternal sample and (**b**) the maternal sample

Physical examination of the patient revealed diffuse, dark brown, tough, bean-sized papules on the face, shark skin-like patches on his back and waist, and patchy hypopigmentation on his limbs and trunk (Fig. 1). Laboratory biochemical tests (blood, urine, stool, liver function, kidney function, electrolyte, coagulation function, blood glucose, and thyroid function) were within the normal range. The result of his G-banding chromosomal analysis was 46XY 22 ps + (Fig. 2a). No abnormality was found in his semen volume, sperm concentration, liquefaction time, motility, and morphology. The clinical diagnosis was TSC and genetic testing was recommended to confirm the clinical diagnosis.

**Fig. 3:**
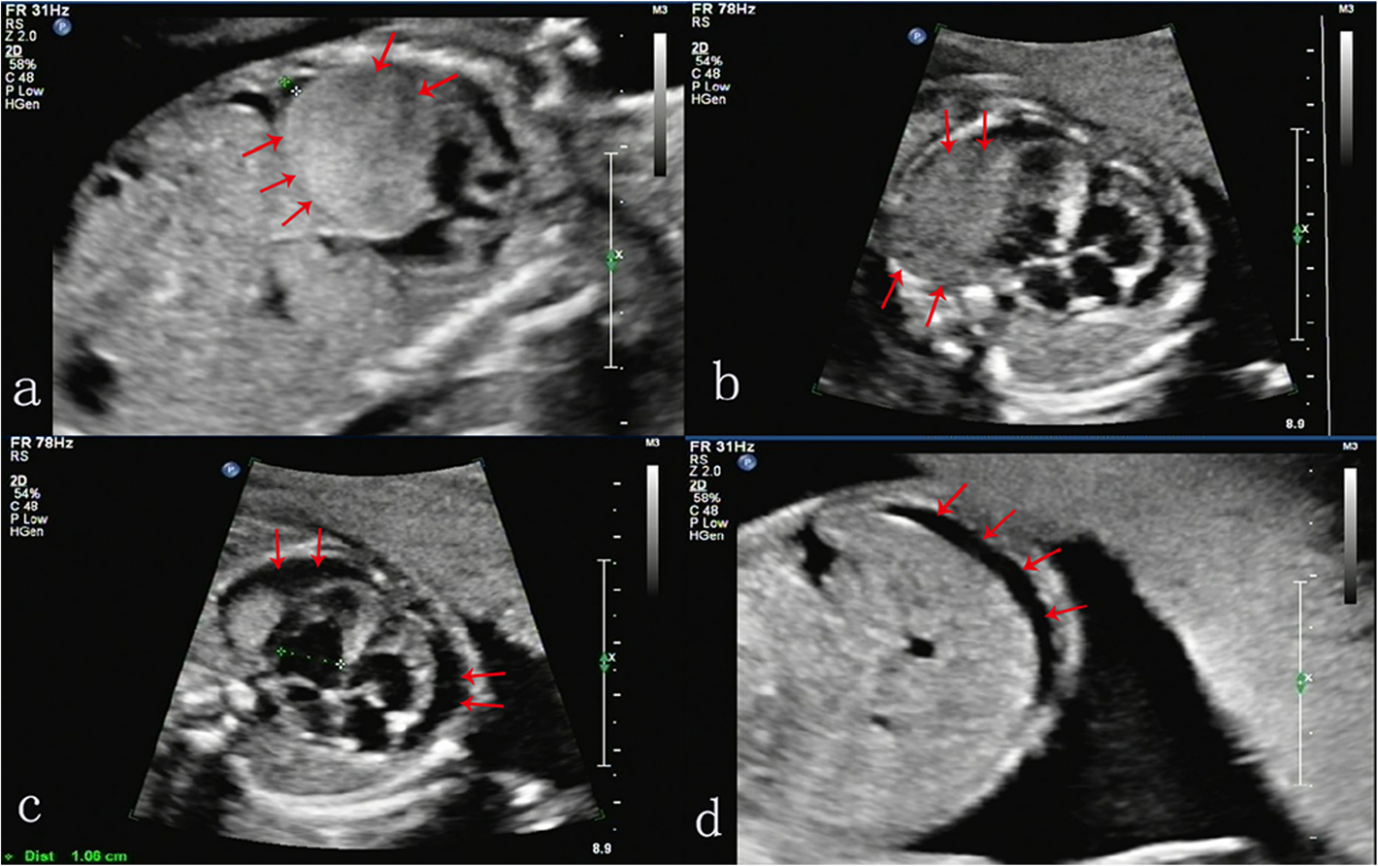
The results of fetal ultrasound showed (**a**, **b**) cardiac rhabdomyosarcoma, (**c**) pericardial effusion, and (**d**) ascites

The male patient’s spouse was healthy. The result of her G-banding karyotype analysis was 46, XX (Fig. 2b). She was married to the patient at the age of 28 and experienced three pregnancies. The first two were miscarriages with arrested intrauterine fetal development at the first trimester of unidentified causes. No genetic analysis were performed on these fetuses. The third pregnancy resulted in induced abortion at 30 weeks into gestation due to “abnormal echogenic nodules in the fetal thoracic cavity and fetal oedema, indicative of cardiac rhabdomyoma, pericardial effusion and ascites” as diagnosed by three-dimensional ultrasonography (Fig. 3). The couple refused fetal autopsy, but the fetal tissue sample was collected.

Peripheral whole blood was collected from the male patient with his informed consent, from which the genomic DNA was extracted and subjected to next-generation sequencing (NGS) by Beijing CIC Clinical Laboratory (Beijing, China) as previously described [4]. The sequencing data were analyzed for point mutations, copy number variations, and chimeric genes in *TSC1* and *TSC2*, the two candidate pathogenic genes of TSC. The average sequencing depth for target genes was 199.65 × .

**Fig. 4:**
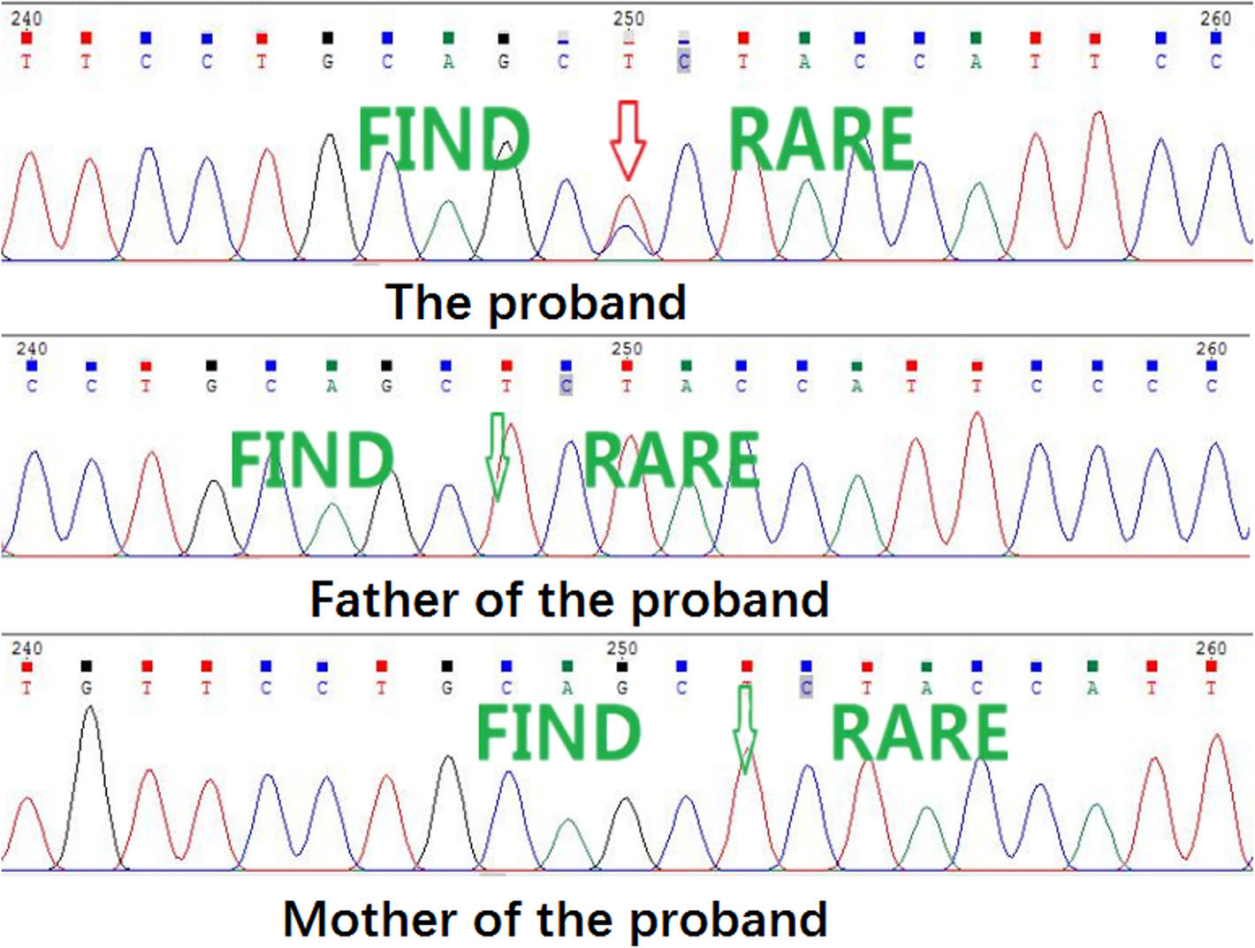
Heterozygous *TSC2*:c.4511 T > C variant (arrow) was identified in the paternal sample (proband) but not in his parents

NGS analysis identified a single-base heterozygous variant of NM_000548.3:exon35:c.4511 T > C:p.L1504P in the paternal sample’s *TSC2* gene (Chr16:2134969, CDS34 gene subregion) (Table 1). We verified this variant in the DNA extracted from the peripheral blood samples of the patient’s parents by Sanger sequencing. The result showed that neither of the male patient’s parents carried this *TSC2* variant (Fig. 4).

**Table 1:** Summary of the novel *TSC2* variant

Gene		*TSC2*
Inheritance		*de novo*
Location		Exon35
Clinical significance		Likely pathogenic [PS2, PM2, PM5, PP3]
*In silico* analysis	Polyphen2	Probably damaging (score:0.999 for sensitivity 0.14 and specificity 0.95)
	Mutation Taster	Disease causing (prob: 0.999);
		Amino acid sequence changed;
		Known disease mutation at this position (HGMD CM091108);
		Protein features (might be) affected;
		Splice site changes
	PROVEAN	Deleterious (score: −5.807)
Population frequency	1000 Genomes	Absence
	ExAC	Absence

Sequencing results were analyzed and compared online with the Geneious Prime software (Geneious Biologics). The *TSC2*:c.4511 T > C:p.L1504P variant has not been described previously. However, another substitution at the same nucleotide (*TSC2*:c.4511 T > A:p.L1504H) has been reported from the HGMD database (http://www.hgmd.cf.ac.uk/ac) to be confirmed as pathogenic (HGMD CM091108). The frequency of this identified variant among the population was analyzed using the 1000 Genomes (http://browser.1000genomes.org/), Exome Aggregation Consortium (ExAC) (http://exac.broadinstitute.org/), and Exome Variant Server (http://evs.gs.washington.edu/EVS) databases. This variant was absent from all databases. PROVEAN (http://provean.jcvi.org/index.php), PolyPhen2 (http://genetics.bwh.harvard.edu/pph2/), and Mutation Taster (http://www.mutationtaster.org/) were used to analyze the effect of this TSC2 gene variation on protein structure and function, which all predicted the TSC2:p.L1504P variant to be deleterious or disease causing with high confidence scores. (Table 1).

**Fig. 5:**
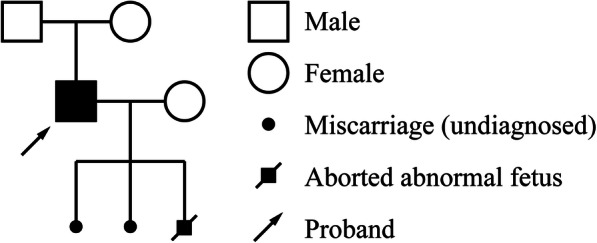
Family pedigree of the case

Then we isolated genomic DNA from the peripheral blood of the patient (paternal sample), his wife (maternal sample), and the tissue sample of the aborted 30-week fetus, and analyzed the specific SNP sites at and near *TSC*2 gene (NM_000548.3 chr16:2097472–2,138,713 forward transcript) using multiplex PCR combined NGS by Beijing Garbo Medical Laboratory Co. LTD (Beijing, China). The sequencing depths were 236×, 212×, and 305× respectively for the paternal sample, maternal sample and aborted fetus. The same heterozygous variant *TSC2*:c.4511 T > C was detected in the aborted fetus, whereas the maternal sample did not harbour this variant. The family pedigree is shown in Fig. 5.

## Discussion and conclusions

In this case, we identified a previous undescribed de novo heterozygous *TSC2*:c.4511 T > C:p.L1504P variant in a TSC patient whose wife had multiple miscarriages and in one aborted abnormal fetus by NGS.

TSC is an autosomal dominant neurocutaneous syndrome with an estimated incidence of 1/6000 to 1/10000 [5]. Sporadic TSC accounts for approximately 2/3 of all cases and family inheritance for approximately 1/3, and there is no difference in prevalence between men and women. The contribution of *TSC1* and *TSC2* variants is similar in inherited genetic cases of TSC, while *TSC2* mutations are 4–5 times more common in sporadic TSC cases. The main types of variant in *TSC1* are point mutations (nonsense, missense, splicing site, frameshift), and deletion/insertion of small fragments [6, 7]. Most of the variants in *TSC2* are missense and nonsense mutations, less frequently small/large fragment deletions, and splice site mutations, which are often accompanied by genetic recombination [8, 9]. According to the LOVD reported mutation database, the ratio of the frequencies of *TSC2* variants vs. *TSC1* variants was approximately 7:3.

Different *TSC1* and *TSC2* variants lead to varied clinical phenotypes. Therefore, an increasing number of studies have focused on the correlation between phenotype and genotype in TSC patients [10, 11]. Curatolo et al. found that the clinical symptoms caused by *TSC2* variants were more serious than those by *TSC1* variants, the former typically included mental retardation, epilepsy, and facial angiofibroma [12]. In this case, the patient presented severe epileptic seizures and facial angiofibroma, which matches the typical manifestation of *TSC2* mutation. As no other variants was identified in the patient’s *TSC1* or *TSC2* gene, it is highly likely that this novel variant caused this sporadic TSC case in the patient.

According to the ACMG/AMP criteria for classifying pathogenic variants [13], this variant was qualified for the following criteria: PS2 (de novo in a patient with the disease and no family history), PM2 (absent from controls in Exome Sequencing Project, 1000 Genomes or ExAC), PM5 (novel missense change at an amino acid residue where a different missense change determined to be pathogenic has been seen before), PP3 (multiple lines of computational evidence support a deleterious effect on the gene or gene product). Although the aborted fetus had been identified with cardiac rhabdomyoma typical of fetal TSC [14], as the patient refused fetal autopsy, we did not have strong evidence to make a definite diagnosis of TSC in the fetus. Therefore this variant was not qualified for the criterion PP1 (co-segregation with disease in multiple affected family members in a gene definitively known to cause the disease). Taken together, this new *TSC2* variant was considered as a “likely pathogenic” variant.

TSC could adversely affect fetal health. Fetal and maternal TSC can be complicated by preeclampsia, intrauterine growth retardation, preterm labour, pre-term premature rupture of membrane, oligohydramnios, polyhydramnios, hydrops, abruption, haemorrhage from rupture renal tumor, renal failure and fetal demise [15]. There has been no study on the rate of spontaneous miscarriage of fetus from paternal TSC. As no genetic tests had been carried out in the patient’s previous two miscarriages, it was unknown whether the spontaneous miscarriages resulted from fetal TSC.

Considering the potential pathogenicity of this variant, in vitro fertilization and embryo transplantation (IVF-ET) and pre-implantation genetic diagnosis (PGD) for TSC are recommended for this patient in the future to prevent fetal TSC. Future pregnancy should also require close monitoring by fetal ultrasound, echocardiography, and MRI imaging.

In conclusion, we identified a sporadic novel variant in *TSC2* gene in association with the TSC phenotype. This will further expand the *TSC* mutation database and improve our understanding of the molecular pathogenesis of TSC. TSC, as a genetic disease, can present clinical manifestations at an early age or even prenatally, which deteriorate as the patient ages. There is no effective treatment for TSC at present. With PGD, it is possible for families with TSC to bear healthy offspring.

## Data Availability

The reference sequence for validation of the c.4511 T > C:p.L1504P variant in the *TSC2* gene was acquired from the NCBI Nucleotide database by using accession number NM_000548.3. The raw sequencing data of NGS are available in the NCBI’s Sequence Read Archive (SRA) with accession number PRJNA659398 (SRR12538044, SRR12538043, SRR12538042, and SRR12538041). The datasets generated and/or analysed during the current study are available in HGMD database (http://www.hgmd.cf.ac.uk/ac), 1000 Genomes database (http://browser.1000genomes.org/), Exome Aggregation Consortium (ExAC) database (http://exac.broadinstitute.org/), Exome Variant Server database (http://evs.gs.washington.edu/EVS), PROVEAN (http://provean.jcvi.org/index.php), PolyPhen2 (http://genetics.bwh.harvard.edu/pph2/), and Mutation Taster (http://www.mutationtaster.org/).

## Abbreviations

TSC: Tuberous sclerosis complex
NGS: Next-generation sequencing
PGD: Preimplantation genetic diagnosis
IVF-ET: In vitro fertilization and embryo transfer
